# Safe-Sex Behavioral Intention of Chinese College Students: Examining the Effect of Sexual Knowledge Using the Theory of Planned Behavior

**DOI:** 10.3389/fpsyg.2022.805371

**Published:** 2022-05-17

**Authors:** Xin Wang, Yuanqing Jin, Mengqin Tian, Qinzi Zhuo, Chien-Liang Lin, Pengfei Hu, Ting Wang

**Affiliations:** College of Science and Technology, Ningbo University, Ningbo, China

**Keywords:** theory of planned behavior, safe sexual behavior, sexual knowledge, college student, sexual education

## Abstract

Numerous contemporary studies have examined safe sexual behavior among college students. In China, families are reluctant to discuss sexual behavior, thus understanding and exploring the sexual knowledge of college students and the promotion of safe sexual behavior is essential. On the basis of the theory of planned behavior (TPB), a cognitive behavioral theory that is widely used to predict human social behavior, we conducted an in-depth investigation of the factors influencing the sexual behavioral intentions of Chinese college students. We referenced the relevant literature to develop a TPB-based model for analyzing differences in sexual knowledge. Our statistical analysis revealed the following: (1) subjective norms and behavior control are key variables that influence the safe sexual behavior of college students; (2) attitudes and safe-sex behavioral intentions are influential in groups with extensive sexual knowledge; (3) behavioral control and subjective norms influence the differences in the comparative sexual knowledge of students. On the basis of the present results, we propose research recommendations and directions for the development of sex education in China.

## Introduction

Sexual health education is a key element in high-quality college education, but it is often neglected. In particular, sexual health education in mainland China has long been conservative, and most parents and teachers explain sex-related topics in a vague manner (Liao and Huang, 2012). Yang et al. (2018) highlighted that the power imbalance between men and women in sexual decision-making persists in China; to address thus imbalance, women must acquire more knowledge about sexuality. Girls are more conservative than boys in receiving sexual knowledge and education. Young boys commonly turn to the Internet for sex-related knowledge because of their conservative environment; Internet media, including pornography, are used as resources for acquiring information on sexuality (Chen et al., 2016). The popularity of the Internet and social media has also exposed young people to diverse cultures from various countries and differing social norms and notions related to sexual health (Trekels et al., 2018). The problems associated with sexual behaviors among college students are also increasing considerably. Zhang et al. (2018) conducted a survey on sex education and sexual behavior among college students and discovered that 16% of college students were already engaging in sexual behaviors. College students are a large and special group, and their involvement in unsafe sexual behaviors exposes them to various risks. A study revealed that less than 30% of college students in mainland China used condoms when having sex for the first time (Lin et al., 2021b). More than 6 million abortions are performed annually on women aged < 25 years, and the proportion of women with multiple partners has reached 48.9% (Liu, 2018). According to the Joint United Nations Programme on HIV/AIDS [UNAIDS] (2013), having multiple sexual partners is a phenomenon that contributes to the increasing rate of human immunodeficiency virus (HIV) infection among college students. Kalolo et al. (2019) reported that unsafe sexual behavior among adolescents is a key obstacle in the fight against HIV transmission. College students who have sex do not take appropriate protective measures because they fear that doing so reduces the pleasure of sexual intercourse and may even threaten the development of their relationships (Strachman and Impett, 2009). Moreover, women under the age of 25 account for almost half of all abortions performed in China annually, and most of these abortions are performed on college students (Lin et al., 2021b). Therefore, to reduce the social problems caused by unsafe sex among college students, Chinese college students’ understanding of safe sex must first be investigated, and the relevant data that are collected may help to popularize sex education in colleges and universities in China. Ganchimeg et al. (2014) suggested that adolescents with unplanned pregnancies who face childbirth-, parenting-, or abortion-related challenges may experience increased physical health problems. Young people represent the future of the country and mankind, thus understanding and improving their sexual health is a key task. With increased education and the corresponding reduction in risky sexual behaviors, problems such as infectious diseases and unwanted pregnancies can be mitigated (Tseng et al., 2020). The findings of the aforementioned studies indicate that understanding the level of awareness of safe sexual behaviors and sexual knowledge among Chinese college students is the key to reducing their unsafe sexual behaviors; this awareness is also essential for Chinese college students to improve their sex-related knowledge.

At the individual level, behavior change theories, such as the theory of planned behavior (TPB; Ajzen, 1991) and theory of reasoned action (TRA; Fishbein and Ajzen, 1975), have been widely applied in public health research and practice to explain and improve human health behaviors (Glanz and Bishop, 2010; Guerin and Toland, 2020). Numerous public health studies have adopted the TPB (Guerin et al., 2018; Siuki et al., 2019; Lin et al., 2021b); the theory is thus appropriate for examining health-care related matters. The TPB is regarded as more appropriate than the TRA for studying behaviors (Montaño and Kasprzyk, 2015; Vafaeenajar et al., 2015). In particular, in the context of psychological and social behaviors, public health and health problems are related to planned behavior (Liang et al., 2017), and numerous researchers have adopted the TPB as their core theoretical framework for discussing safe-sex behavioral intentions. Furthermore, because the TPB can effectively explain human behavioral intentions, it has strong explanatory power for making behavioral predictions (Liang et al., 2017; Khani Jeihooni et al., 2019; Tseng et al., 2020; Lin et al., 2021b). A study of Tseng et al. (2020) also suggested that the predictive power of the TPB model for behavioral intentions can be enhanced by identifying extended constructs. Therefore, considering the crucial role of the safe-sex behavioral intentions of college students, the present study used sexual knowledge as an extended variable for explaining whether differences in Chinese college students’ level of sexual knowledge affect their safe-sex behavioral intentions. The following two research questions were examined in the present study:

1. Do college students’ attitudes, subjective norms, and behavioral control in relation to safe sexual behaviors affect their safe-sex behavioral intentions?
2. Do differences in the level of understanding of sexual knowledge among college students have different effects on their behavioral intentions?

## Literature Review

### Theory of Planned Behavior

Researchers examining personal behavior have previously suggested that human behavior is rational and volitional (Fishbein and Ajzen, 1975). However, human behavior is not only affected by the individual will but also by factors such as individual ability, environment, and conditions. Therefore, Ajzen (1991) further expanded the variables of the TRA and added the variable of perceived behavioral control to develop the TPB. Following the establishment of the TPB, numerous studies have adopted it to explain and predict behavioral problems related to individual sexual health (Liang et al., 2017; Tseng et al., 2020; Lin et al., 2021b). The TPB focuses specifically on the effective social factors and personal attitudes of people in behavioral development (Eaton and Stephens, 2019); this is because the ultimate prediction of the TPB is individual intention. However, intentions are successively influenced by three factors, namely a person’s attitude toward a specific behavior, their subjective norms, and their perceived behavioral control (Sharma and Romas, 2011; Rogers, 2017). In a study by Moeini et al. (2017), the TPB was applied to promote safe sexual behavior among drug addicts, and the results indicated that the effective promotion of sex education can increase the awareness of safe sexual behavior among drug addicts. Armitage and Talibudeen (2010) provided evidence that interventions based on the TPB can successfully alter the safe-sex behavioral intentions of adolescents. Other studies have used attitude, subjective norms, and perceived behavioral control as the predictors in their TPB models, revealing that this method can successfully predict some safe sexual behaviors (Armitage and Talibudeen, 2010; Khani Jeihooni et al., 2019). Numerous studies have also added exogenous variables to increase the predictive validity of their models. For example, Bryan et al. (2002) added biological sex to their TPB model to explore the willingness of college students to use condoms. Cheng and Huang (2018) added sexual orientation and gender to assess how the TPB can be applied to influence condom use. Tseng et al. (2020) discovered that an extended TPB model can effectively predict safe sexual behaviors and added external variables that increased the predictive validity of the model. They also discovered that the extended TPB model can successfully predict the safe-sex behavioral intention of female adolescents; the model was also revealed to empower young women to establish a subjective consciousness and recognize their maturity and physical autonomy, which are crucial for their sexual health (Tseng et al., 2020). Conner and Flesch (2001) investigated the intention of British college students to engage in occasional sexual intercourse; they reported that attitude, subjective norms, and behavioral control predicted intention to engage in occasional sexual behavior, and other external variables, such as past behavior, biological sex, drinking, and the use of condoms can strengthen their ability to predict behavior. By contrast, Lin et al. (2021b) added basic information as moderator variables (e.g., biological sex and sexual experience) in their TPB model, and they reported that gender and sexual experience have different influences on safe-sex behavioral intention. Overall, the extended TPB architecture has greater predictive power than the original TPB for explaining safe sexual behavior (Turchik and Gidycz, 2012). The findings of the aforementioned studies highlight the key role of the extended TPB model in explaining the safe sexual intention of college students. Therefore, in the subsequent literature review, we considered the role of sexual knowledge in safe sexual behavior.

### Sexual Knowledge

The term sexual knowledge refers to the individual knowledge and awareness of sex and sexual behavior (including physiological aspects, reproduction, performance, and individual sexual behavior; Soltani et al., 2017). The lack of sexual knowledge causes numerous problems, such as abusive sexual activities, inappropriate sexual roles, and unsafe sexual behavior (Salimi and Fatehizadeh, 2013). However, studies have mainly divided the concept of sexual knowledge into five components, namely general knowledge, oral contraceptive use, condom use, knowledge of chlamydia, and knowledge of HIV/AIDS (Langille et al., 1998). However, in today’s digital era, people can access a range of information through the Internet, incorrect sexual knowledge are disseminated on various platforms, and sexual knowledge originates not only from parents or teachers but also from various media channels (Lou et al., 2012). Although the sources of sexual knowledge are diverse, the acquisition of incorrect information by college students also causes them to engage in inappropriate behaviors. Unscreened information may have an undesired influence; in addition to premarital sex, unsafe sex can also lead to unwanted pregnancies, abortions, single-parent families, and the spread of sexually transmitted diseases (e.g., HIV/AIDS), all of which have a considerable effects on the physical and mental health of people (Deligeoroglou et al., 2006). A study also reported that soon to be married women experience sexual anxiety when they lack sufficient sexual knowledge; however, the study also indicated that relevant education or knowledge dissemination can effectively enhance these women’s sexual knowledge and reduce their premarital sexual anxiety (Hemati et al., 2020). In China, sexual health education for college students is highly conservative. Many college students avoid discussing sex-related topics, and most parents do not provide effective sexual health education (Liao and Huang, 2012). Specifically, the lack of sexual knowledge and limited adoption of safe sex are two key reasons for the increase in HIV infection rate among college students. Therefore, a lack of knowledge of venereal diseases among college students can increase the transmission of venereal diseases or HIV, which has a serious effect on the physical and mental health of these students and their future studies, work, and lives (Tang et al., 2011; Li et al., 2013). Therefore, in the dissemination of sexual knowledge and implementation of health education, knowledge of sexual physiology and common sexual diseases should be emphasized. Students can only consciously protect themselves and prevent the transmission of sexual diseases if they acquire the relevant knowledge of sexual diseases (Wang et al., 2010). Among the studies that have explored sexual knowledge in the context of Chinese universities, few have applied systematic or scientific behavioral theories to explain Chinese college students’ understanding and cognition of sexual knowledge. Therefore, we applied a TPB model and used various background terms to explore the effects of sexual knowledge on behavioral intention.

## Research Model and Hypotheses

### Research Model

**FIGURE 1:**
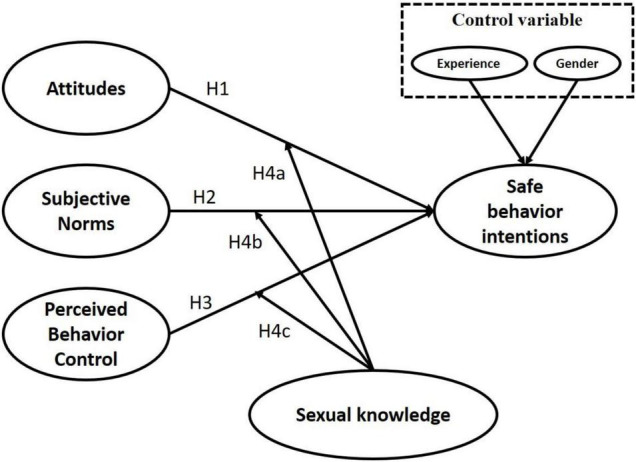
Research model.

We adopted the TPB (Ajzen, 1991) as our core theory. We used attitude toward safe sexual behavior, subjective norms, and perceived behavioral control as the main influence variables; and added sexual knowledge as an extended variable that affects safe sexual behavior through the establishment of the aforementioned hypotheses. The model of the present study was based on the extended TPB, and it was used to predict the safe-sex behavioral intention of college students. The research model is illustrated in Figure 1.

### Hypotheses

The TPB is a basic theory that is widely applied to explain the intention of people to engage in a specific behavior. The TPB can be used to analyze the factors affecting intention, and it is particularly useful for predicting the behavioral intention of people; thus, researchers can construct a theoretical concept that is tailored to the research situation and personal or social factors (Liñán et al., 2016; Morales et al., 2018). Therefore, this theory is widely applied in various research contexts. In the TPB, three variables affect intention, namely perceived behavioral control (an individual’s evaluation of behavior that they intend to perform on the basis of their own ability or difficulties), attitude (an individual’s personal positive or negative beliefs about a specific behavior or action), and subjective norms (how an individual’s feelings about an act is influenced by the people around them or influential people such as their parents, friends, or colleagues), all of which can directly predict an individual’s intention to act (Lee, 2010; Liñán et al., 2016; Robertsen et al., 2018; Al-Jubari et al., 2019; Canova et al., 2020; Su et al., 2021). Numerous studies have also used the TPB to explain the intentions of students in relation to safe sexual behaviors, such as a study that examined the implementation of sex education by nursing students (Sharon et al., 2020). Turchik and Gidycz (2012) studied the risky sexual behavior of students, Morales et al. (2018) examined the spread of sexually transmitted diseases among young people, and Tseng et al. (2020) discussed the safe-sex behavioral intention of adolescents. On the basis of studies in which the TPB model and architecture was applied to discuss safe-sex behavioral intention, the following hypotheses were proposed:

H1: The attitudes of college students toward sexual behavior would have a significant influence on their safe-sex behavioral intention.
H2: The subjective norms of college students regarding sexual behavior would have a significant influence on their safe-sex behavioral intention.
H3: The perceived behavioral control of college students over sexual behavior would have a significant influence on their safe-sex behavioral intention.

Previous studies have demonstrated that gender and level of sexual experience influence the safe sexual behavior of students (Morales et al., 2018; Sharon et al., 2020; Lin et al., 2021b). Lo and Huang (2018) reviewed the literature and discovered that different levels of understanding of sexual knowledge leads to differences in the intention of college students to engage in safe sexual behavior. Therefore, the present study made the practical inference that college students have varying levels of sexual knowledge that lead to differences in their cognition of safe sexual behavior. On the basis of the aforementioned discussion, we proposed the following hypotheses about safe-sex behavioral intention:

H4a: Differences in the sexual knowledge of college students would have a significant influence on their sexual attitude and safe-sex behavioral intention.
H4b: Differences in the sexual knowledge of college students would have a significant influence on their subjective norms and safe-sex behavioral intention.
H4c: Differences in the sexual knowledge of college students would have a significant influence on their perceived behavioral control and safe-sex behavioral intention.

### Construct Operationalization

The items on the questionnaire employed in the present study were developed and adjusted by referencing the literature and considering the setting of the present study. The questionnaire items were mainly based on the TPB scale used by Turchik and Gidycz (2012) and Lin et al. (2021b), and all the items were scored using a 7-point Likert scale. The items were focused on measuring four constructs, namely attitude, subjective norms, perceived behavioral control, and intention to engage in safe sexual behavior. Seven, six, nine, and eighteen items were used to measure the perceived behavioral control, subjective norm, safe-sex behavioral intention, and sexual behavior attitude constructs, respectively. The alpha values of all the constructs were greater than 0.7 after referencing the literature (Lin et al., 2021b). The items for assessing sexual knowledge were based on the scale used by Langille et al. (1998). A total of 29 questions were used to assess five domains of sexual knowledge, namely general knowledge (six questions), oral contraception use (four questions), condom use (six questions), knowledge of chlamydia (seven questions), and knowledge of HIV/AIDS (six questions). A correct answer was worth 1 point, and no point was awarded for a wrong answer. The alpha value of the scale was 0.77. Because the items used in the present study were derived from English-language scales, they had to be adapted to the context of universities in China. To this end, the questionnaire was first translated by a professor and two assistant professors of psychology, after which a preliminary test was conducted to ensure that the students who participated in the study could comprehend the questions. Subsequently, the translated Chinese questions were backtranslated into English by a translator who received specialized English language training; this backtranslation step was performed to ensure the accuracy of the questionnaire items.

### Data Collection

The present study focused on the safe-sex behavioral intentions of Chinese college students. Because of the COVID-19 pandemic and related data collection limitations, we used an online questionnaire. We selected the online questionnaire format because it enables convenient and low-cost data collection, quick responses, and access to a wide range of users (Bhattacherjee, 2002). Because of the specificity of the studied topic and to ensure a high response rate, we implemented convenience sampling. The survey data were collected through WJX^1^. In total, 542 questionnaires were collected through the online questionnaire. Questionnaires were excluded under the following conditions (Lin et al., 2021a): responses under a single construct that were all scored identically (e.g., a score of 1 or 7 for all relevant items) or responses with extreme values; (2) a questionnaire submitted from an Internet protocol address that was identical to that of a previous submitted questionnaire (i.e., duplicated questionnaires are excluded); (3) a questionnaire that was completed too quickly (typical completion time was 5–8 min; thus, questionnaires that were completed in less than 3 min were regarded as invalid). We excluded 35 invalid questionnaires and used 507 valid questionnaires for formal data analysis. In total, 201 male students and 306 female students submitted valid responses. Among the respondents, 82 were aged 18 years, 102 were aged 19 years, 129 were aged 20 years, 79 were aged 21 years, 75 were aged 22 years, and 40 were aged 23 years or older. Among them, 209 had never been in a romantic relationship, 160 had previously experienced romantic relationships, and 138 were in romantic relationships at the time of the investigation. Eighty-six students reported having had sex before, whereas 421 students had no such experience. The respondents comprised a greater proportion of male respondents than of female respondents. Study participation was voluntary, and men were generally more willing than women to complete the questionnaire. Relevant studies have indicated that behavioral intentions are influenced by individual factors, such as gender and experience (Gefen et al., 2003; Lin et al., 2021a). Therefore, to reduce the influence of basic information variables on the intention to have safe sex, we measured gender and experience as control variables.

## Results

Partial least squares structural equation modeling (PLS-SEM) was adopted as the main analytical tool for empirical analysis in the present study. The main reasons for adopting SmartPLS are as follows: (1) PLS-SEM is a predictive causality method with strong predictive value when solitary statistical models are used to predict the explanatory power of causal relationships. (2) Compared with traditional covariance-based structural equation modeling, PLS-SEM requires fewer samples to estimate more complex models. (3) In the field of management and psychology, PLS-SEM has been widely applied in social science studies spanning various disciplines (Sarstedt et al., 2017; Hair et al., 2019; Khan et al., 2019). In addition, the questionnaire was designed to reduce common method variance (CMV); this was achieved by applying a split treatment for the constructs in the present study, which allows for fillers to be buffered when switching pages and reduces the effect of the CMV from a measure when a questionnaire is being completed (Podsakoff et al., 2003).

### Measurement Model

The reliability and validity of the measurement models used in the present study were analyzed to ensure that the constructs were sufficiently differentiated (Hair et al., 2017). The assessment of internal consistency was conducted using composite reliability and Cronbach’s α, and the results indicated that the present study met the suggested threshold (Hair et al., 2017). For reliability, the factor loading value should be greater than 0.7 (Hair et al., 2017); the factor loading range in the present study was between 0.777 and 0.960), which indicated favorable internal consistency. Henseler et al. (2014) defined the standardized root mean square residual (SRMR) as the square root of the sum of the squared differences between a model and an empirical correlation matrix, and a value of less than 0.10 indicates a good fit. The overall SRMR of the model used in the present study was 0.064, indicating that the model was acceptable. For construct validity, an average variance extracted of more than 0.5 is recommended (Fornell and Larcker, 1981). The structural model of the present study was evaluated by conducting a variance inflation factor covariance test, and the constructs had values ranging from 1.063 to 1.327, which were all lower than the suggested value of 5 (Hair et al., 2017). Table 1 presents the results of the aforementioned indicators and reveals that they all met the threshold value recommended in the literature.

**TABLE 1:** Factor loading, Cronbach’s α, composite reliability, and average variance extracted.

Construct	Items	Factor loading	Cronbach’s α	Composite reliability	Average variance extracted (AVE)
Subjective norms	SN1 SN2 SN3 SN4 SN5 SN6	0.849 0.884 0.91 0.912 0.857 0.875	0.943	0.954	0.777
Behavioral intention	ITU1 ITU2 ITU3 ITU4 ITU5 ITU6 ITU7 ITU8 ITU9	0.861 0.778 0.857 0.898 0.89 0.923 0.922 0.791 0.777	0.955	0.961	0.735
Attitude	AT1 AT2 AT3 AT4 AT5 AT6 AT7 AT8 AT9 AT10 AT11 AT12 AT13 AT14 AT15 AT16 AT17 AT18	0.835 0.858 0.831 0.891 0.865 0.865 0.898 0.906 0.881 0.913 0.902 0.893 0.876 0.881 0.857 0.896 0.88 0.87	0.983	0.984	0.771
Perceived behavioral control	PBC1 PBC2 PBC3 PBC4 PBC5 PBC6 PBC7	0.796 0.93 0.926 0.955 0.96 0.877 0.866	0.962	0.969	0.815

Discriminant validity primarily tests the degree to which the items used to measure different constructs are discriminated. According to Chin (1998), for a construct to have sufficient discriminant validity, the square root of the mean sampled variance must be greater than the correlation coefficient between constructs. Table 2 presents the matrix of the correlation coefficients between the constructs; in the table, the square root of the mean sampled variance corresponds to the diagonal line, and the remainder of the values are used to test the discriminant validity of the correlation coefficients between the constructs (for the variables and the average variance among the constructs). The results indicated that the square root of the mean sampled variance for each construct was greater than the correlation coefficient between the constructs; thus, the constructs all had discriminant validity (Table 2). Researchers have proposed use of the heterotrait–monotrait ratio (HTMT) as an indicator for testing discriminant validity; Henseler et al. (2015) suggested that a reference value of less than 0.9 between constructs indicates favorable discriminant validity. In the present study, the highest HTMT value was only 0.661 (Table 3); thus, the proposed model had favorable discriminant validity.

**TABLE 2:** Analysis of discriminant validity (Fornell–Larcker criterion).

	Subjective norm	Attitude	Behavioral intention	Perceived behavioral control
Subjective norms	0.881			
Attitude	0.118	0.878		
Behavioral intention	0.53	0.217	0.857	
Perceived behavioral control	0.457	0.283	0.641	0.903

**TABLE 3:** Analysis of discriminant validity (heterotrait–monotrait ratio).

	Subjective norm	Attitude	Behavioral intention	Perceived behavioral control
Subjective norm				
Attitude	0.117			
Behavioral intention	0.548	0.226		
Perceived behavior control	0.477	0.286	0.661	

### Structural Model

**FIGURE 2:**
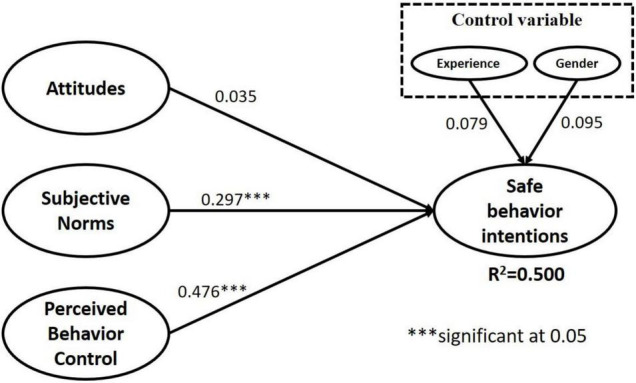
Partial least squares results.

In the present study, we used the bootstrapping method, which is a resampling method used by Hair et al. (2017), to evaluate our PLS results (Figure 2). The sampling was performed with 5,000 resamples, and the results of the structural model analysis are presented in Figure 2. An overall explanatory power of 50% was observed, indicating that the model used in the present study had favorable explanatory power. The empirical results of the H1, H2, and H3 tests provided support for H2 and H3. Specifically, the TPB-based hypotheses that the subjective norms (H2; ß = 0.300; *p* < 0.001) and behavior control (H3; ß = 0.476; *p* < 0.001) of college students would have significant effects on their sexual behavioral intentions were supported; the path coefficient of behavioral control (H3) was revealed to be as high as 0.476. For the effect of attitude (H1; ß = 0.035) on sexual behavioral intention, a non-significant relationship was detected. Finally, among the control variables, sexual experience (ß = 0.079) and gender (ß = 0.095) were revealed to have non-significant influences on the intention to have safe sex.

For H4a to H4c, PLS-SEM multiple group analyses were performed to compare the differences between two samples in terms of the path coefficients of the study model. The significance of the differences in path coefficients was assessed per the method suggested by Cheah et al. (2019). First, the path coefficients of the study model were obtained from two sets of data, and a *t* test was conducted to determine if the two sets of path coefficients were significantly different. Through the aforementioned steps, the difference between the path coefficients of safe-sex behavioral intentions and the level of sexual knowledge was obtained. The results pertaining to extensive sexual knowledge (Table 4) indicated that only the path coefficient of the effect of behavioral control (H4c) on behavioral intentions was significantly different between the two groups (−0.24*), and the path coefficient of poor sexual knowledge (0.566^***^) was greater than that of extensive sexual knowledge. The students with poor sexual knowledge tended to consider their behavioral ability; by contrast, for the students with extensive sexual knowledge, their understanding of their own ability was influenced by their attitudes and subjective norms. The effect of attitude on behavioral intentions was significant for the students with extensive sexual knowledge (0.18^***^) but non-significant for the students with poor sexual knowledge (0.022); and the difference in the path coefficients between the two groups was significant. The aforementioned results indicated that attitudes toward safe-sex behavior affected safe-sex behavioral intentions among the students with extensive sexual knowledge but not among the students with poor sexual knowledge.

**TABLE 4:** Multiple group analysis through partial least squares method (sexual knowledge).

Hypotheses	Relationship	Path coefficient for extensive sexual knowledge	Path coefficient for poor sexual knowledge	Difference between path coefficients	*p* value
H4a	Attitude—> Behavioral intention	0.180***	0.022	0.159	0.068*
H4b	Subjective norm—> Behavioral intention	0.236***	0.344***	−0.108	0.349
H4c	Perceived behavior control—> Behavioral intention	0.326***	0.566***	−0.24	0.056*

*p < 0.1; **p < 0.05; and ***p < 0.01. Notes t = 1.96.

## Discussion and Conclusion

### Discussion

The present study focused on the safe-sex behavioral intentions of Chinese college students. The results indicated that the TPB has favorable predictive power, which is consistent with the findings of numerous studies (Tseng et al., 2020; Lin et al., 2021b). Attitudes toward safe sexual behavior (H1) was revealed to have a non-significant effect on behavioral intentions, which is inconsistent with the findings of other studies (Khani Jeihooni et al., 2019). Our results indicated that a key factor is the entrenched conservative nature of sex education in China; thus, college students still have conservative perceptions of sex education and are reluctant to express their views (Lin et al., 2021b). Therefore, we suggest that schools and families actively collaborate to develop a high-quality and sound sex education curriculum that helps college students to develop appropriate sexual values. Subjective norms (H2) and behavioral control (H3) were revealed to affect safe-sex behavioral intentions, which is consistent with the findings of another study (Wang et al., 2019). Therefore, to enhance the education on safe sexual behavior for college students, the families and friends of these students must be a focus. Furthermore, in the Internet era, most students acquire their sexual knowledge not only through textbooks but also through other sources; to prevent the dissemination of incorrect sexual knowledge, comprehensive knowledge and ability should become key areas of focus in sex education. The present study also revealed a significant difference between attitude (H4a) and behavioral control (H4c) in terms of their effects on behavioral intentions; by contrast, no significant difference was detected for subjective norms (H4b). Essentially, college students lack sexual knowledge or have a poor understanding of sexual knowledge, mainly because of their deep-rooted conservative beliefs. Moreover, inadequate teaching tools and the lack of teachers and information caused by the urban–rural gap further exacerbate the problem of insufficient sexual knowledge (Jin et al., 2008). Studies have highlighted the problem of insufficient sexual knowledge among college students from rural areas; although their research results were not obtained through TPB-based models, some of their conclusions are similar to those of the present study (Li et al., 2013; Lyu et al., 2020). Therefore, a suggested explanation for the gap in sexual knowledge among Chinese college students is the deep-rooted conservative culture in China; in particular, the health education in China rarely conveys information about sexual behavior–related problems such as the phenomenon of single mothers (Chen et al., 2008). In a study conducted in the United States, Swenson et al. (2010) surveyed African-American teenagers and reported that a correct understanding of sexual knowledge helps teenagers to acquire the correct sexual values and consciousness. Oman et al. (2018) also investigated the sexual knowledge, attitudes, and behaviors of youths in the United States, and their results indicated that a greater understanding of sexual knowledge reduces the influence of incorrect values and increases the likelihood of positive sexual ethics being practiced.

### Practical and Theory Implications

In general, the difference in sexual knowledge among college students can still be attributed to the implicit nature of sex education in China. Therefore, schools should provide elective sex education courses to help students acquire systematic, comprehensive, and scientific knowledge about sex in a formal classroom setting, where sexual morality and appropriate sexual concepts can be established, and students can be taught methods for identifying and preventing sexually transmitted diseases. This strategy is similar to those proposed in studies conducted outside of China (Li, 2020; Lin et al., 2021b). For curriculum design, teaching materials that are certified by experts or the relevant mental health curriculum development committees (formed by academic assistants) should be used to examine and verify curriculum standards, and curriculum design should also be improved to incorporate current trends. Furthermore, sex education curricula should be constructed by teachers who majored in psychology and sociology, and only suitably qualified individuals should teach sex education. Curricula should be designed in accordance with local circumstances and current trends. Schools should help students develop a scientific attitude toward sex by promoting and holding on-campus lectures on sexual knowledge. In addition, they should establish comprehensive counseling centers and dedicated websites that students can access anonymously and submit queries to; teachers with the appropriate professional training can then answer these queries online, such that students who are confused about sex can receive adequate advice. Sex education would benefit not only from curriculum improvement but also from increased support from government departments. Therefore, government units should focus on improving the sex education curriculum and enhancing the counseling mechanism and media publicity of universities, especially for the periods before and after school vacations; they should also strengthen efforts to publicize the crucial role of safe sex. Policies can be implemented to encourage universities to establish sex education zones or gender equality committees to disseminate sexual knowledge and the crucial role of safe sex. For example contraceptive measures such as the use of condoms can be promoted, knowledge regarding the transmission routes of HIV can be disseminated, and counseling mechanisms related to gender equality can be established; these measures can effectively reduce the transmission of sexual diseases and promote the crucial role of safe sex.

Although studies have verified that sexual knowledge is a key concept for understanding sexual behaviors (Tang et al., 2011; Oman et al., 2018; Maes et al., 2020), no study has used the TPB framework to examine sexual knowledge. Therefore, the present study integrated the concept of sexual knowledge into the framework of the original TPB, thereby contributing to the literature on the application of the original TPB to explain the intention to have safe sex.

### Limitations and Future Research

The present study used an online questionnaire to investigate the safe-sex behavioral intentions of Chinese college students, and this is a method with several limitations. First, the sample was obtained mainly through convenience sampling, and the sampling area was primarily the universities in Zhejiang, China. The lack of samples from schools in other provinces of China could have introduced biases relating to the understanding of sex education. Therefore, our results may only be generalized to developed Chinese provinces. Second, the present study was based only on the TPB. To address this shortcoming, the analysis of the differences in sexual knowledge and their effects on our results can be expanded to allow for greater diversity in the selection of future variables (e.g., sexual experience, sensation seeking, and exposure to sexually explicit materials). In particular, gender-, ethnicity-, and province-related differences can be explored to generate more substantive suggestions in relation to our results. Finally, the present study collected quantitative data through a survey and did not employ qualitative methods or other research methods to enhance the strength of the findings. Therefore, mixed-method studies should be conducted to identify the key influences that affect the safe sexual behaviors of college students.

## Data Availability Statement

The original contributions presented in the study are included in the article/supplementary material, further inquiries can be directed to the corresponding author/s.

## Author Contributions

XW, YJ, and C-LL designed the research and provided guidance throughout the entire research process. MT, QZ, and PH collected the references, did the literature analysis, and wrote the manuscript. XW, MT, TW, and QZ helped with translation and offered modification suggestions. C-LL, YJ, and PH participated in the literature collection, analysis, and organization. All authors listed have made a substantial, direct, and intellectual contribution to the work, and approved it for publication.

## Conflict of Interest

The authors declare that the research was conducted in the absence of any commercial or financial relationships that could be construed as a potential conflict of interest.

## Publisher’s Note

All claims expressed in this article are solely those of the authors and do not necessarily represent those of their affiliated organizations, or those of the publisher, the editors and the reviewers. Any product that may be evaluated in this article, or claim that may be made by its manufacturer, is not guaranteed or endorsed by the publisher.

## Footnotes

1
https://www.wjx.cn/
